# Cross-country variability in Mediterranean lifestyle adherence and its psychosocial and behavioural correlates: insights from the MEDIET4ALL project across 10 Mediterranean and neighboring countries

**DOI:** 10.3389/fnut.2026.1807414

**Published:** 2026-04-15

**Authors:** Achraf Ammar, Atef Salem, Slim Zarzissi, Tania Abril-Mera, Khaled Trabelsi, Bassem Bouaziz, Mohamed Ali Boujelbane, Mohamed Kerkeni, Liwa Masmoudi, Juliane Heydenreich, Christiana Schallhorn, Gabriel Müller, Ayse Merve Uyar, Hadeel Ali Ghazzawi, Adam Tawfiq Amawi, Bekir Erhan Orhan, Giuseppe Grosso, Osama Abdelkarim, Tarak Driss, Kais El Abed, Wassim Moalla, Piotr Zmijewski, Frédéric Debeaufort, Nasreddine Benbettaieb, Clément Poulain, Laura Reyes, Amparo Gamero, Marta Cuenca-Ortolá, Antonio Cilla, Nicola Francesca, Concetta Maria Messina, Enrico Viola, Björn Lorenzen, Stefania Filice, Sadjia Lahiani, Taha Khaldi, Nafaa Souissi, Omar Boukhris, Evelyn Frias-Toral, Haitham Jahrami, Waqar Husain, Walid Mahdi, Nizar Souissi, Hamdi Chtourou, Wolfgang I. Schöllhorn

**Affiliations:** 1 Department of Training and Movement Science, Institute of Sport Science, Johannes Gutenberg-University Mainz, Mainz, Germany; 2 Research Laboratory, Molecular Bases of Human Pathology, LR19ES13, Faculty of Medicine of Sfax, University of Sfax, Sfax, Tunisia; 3 Interdisciplinary Laboratory in Neurosciences, Physiology, and Psychology, Physical Activity, Health, and Learning (LINP2), UFR STAPS, Paris Nanterre University, Nanterre, France; 4 Department of Nutrition and Food Technology, School of Agriculture, The University of Jordan, Amman, Jordan; 5 High Institute of Sport and Physical Education of Sfax, University of Sfax, Sfax, Tunisia; 6 School of Medicine, Universidad Católica de Santiago de Guayaquil, Guayaquil, Ecuador; 7 Research Laboratory, Education, Motricity, Sport and Health, EM2S, LR19JS01, High Institute of Sport and Physical Education of Sfax, University of Sfax, Sfax, Tunisia; 8 Department of Movement Sciences and Sports Training, School of Sport Science, The University of Jordan, Amman, Jordan; 9 Multimedia InfoRmation Systems and Advanced Computing Laboratory (MIRACL), University of Sfax, Sfax, Tunisia; 10 Higher Institute of Computer Science and Multimedia of Sfax (ISIMS), University of Sfax, Sfax, Tunisia; 11 Department of Experimental Sports Nutrition, Faculty of Sports Sciences, Leipzig University, Leipzig, Germany; 12 Department of Sports Economics, Sociology and History, Institute of Sport Science, Johannes Gutenberg-University Mainz, Mainz, Germany; 13 Faculty of Sports Sciences, Istanbul Aydın University, Istanbul, Türkiye; 14 Department of Biomedical and Biotechnological Sciences, University of Catania, Catania, Italy; 15 Department of Sports Management, School of Business, ESLSCA University, Giza, Egypt; 16 Institute of Sports and Sports Science, Karlsruhe Institute of Technology, Karlsruhe, Germany; 17 Faculty of Sport Sciences, Assiut University, Assiut, Egypt; 18 Department of Biochemistry, Gdansk University of Physical Education and Sport, Gdansk, Poland; 19 Department BioEngineering, Institut Universitaire de Technologie IUT-Dijon-Auxerre-Nevers, University Burgundy Europe, Dijon, France; 20 Joint Research Unit UMR PAM-Food Processing and Microbiology, Université Bourgogne Europe, INRAE, Institut AgroDijon, Dijon, France; 21 Vitagora Innovation Cluster, Dijon, France; 22 Department of Preventive Medicine and Public Health, Food Science, Toxicology and Forensic Medicine, Faculty of Pharmacy and Food Sciences, University of Valencia, Valencia, Spain; 23 Department of Agricultural Food and Forest Sciences SAAF, University of Palermo, Palermo, Italy; 24 Laboratory of Marine Biochemistry and Ecotoxicology, Department of Earth and Marine Sciences DiSTeM, University of Palermo, Trapani, Italy; 25 Microtarians SIS, Société d’Impact Sociétal, Luxembourg, Luxembourg; 26 VALCORE Laboratory, Department of Biology, Faculty of Science, University of M’hamed Bougara Boumerdes, Boumerdes, Algeria; 27 Biotechnology Research Center C.R.Bt Constantine ALGERIA, Constantine, Algeria; 28 Research Unit, Physical Activity, Sport, and Health, UR18JS01, National Observatory of Sport, Tunis, Tunisia; 29 SIESTA Research Group, School of Allied Health, Human Services and Sport, La Trobe University, Melbourne, VIC, Australia; 30 Sport, Performance, and Nutrition Research Group, School of Allied Health, Human Services and Sport, La Trobe University, Melbourne, VIC, Australia; 31 Escuela de Medicina, Universidad Espíritu Santo, Samborondón, Ecuador; 32 Division of Research, Texas State University, San Marcos, TX, United States; 33 Government Hospitals, Manama, Bahrain; 34 Department of Psychiatry, College of Medicine and Medical Sciences, Arabian Gulf University, Manama, Bahrain; 35 Department of Humanities, COMSATS University Islamabad, Islamabad, Pakistan

**Keywords:** behavioural epidemiology, population-based survey, multidimensional lifestyle, health behaviours, mental health, physical activity patterns, social participation, sleep health

## Abstract

**Background:**

The Mediterranean lifestyle is increasingly recognized as a multidimensional determinant of health. However, cross-country comparisons using harmonized instruments remain limited. This study aimed to provide a comprehensive country-by-country comparison of Mediterranean lifestyle adherence and associated psychosocial and lifestyle correlates across 10 Mediterranean and neighboring countries participating in the MEDIET4ALL project.

**Methods:**

Cross-sectional data were collected from 4,010 participants (age: 37.2 ± 15.4 years; 59.5% female) using the multinational MEDIET4ALL e-survey. Mediterranean lifestyle adherence was assessed using the MedLife Index and its three domains. Psychosocial status, sleep characteristics, physical activity, sedentary behaviour, social participation, and technology use were evaluated using validated instruments.

**Results:**

Significant cross-country differences were observed in global MedLife adherence and across all domains (*p* < 0.001, *η*^2^ = 0.07–0.11), as well as in the distribution of adherence categories across countries (*χ*^2^ = 113.936, *p* < 0.001). Spain consistently showed higher MedLife scores than several countries (*z* = 3.42–8.12, adjusted *p* < 0.001–0.02) and tended to display higher proportions of participants in the high-adherence category, whereas lower adherence was observed in multiple non-Mediterranean and North African contexts. Psychological distress differed significantly between countries (*p* < 0.001), with several contexts showing elevated depression, anxiety, or stress levels (*z* ≈ 3.57–14.29, adjusted *p* < 0.001–0.05). Life satisfaction and social participation also varied substantially (194.86, *p* < 0.001), with some European countries reporting lower social participation compared with Mediterranean and neighboring contexts (*z* = 3.79–9.31, adjusted *p* < 0.001–0.05). Sleep parameters and insomnia severity differed markedly across countries (*H* = 66.64–198.63, *p* < 0.001), with less favourable sleep profiles observed in several contexts (*z* ≈ 3.28–12.82, adjusted *p* < 0.001–0.05). Physical activity and sedentary behaviour showed pronounced variability (*p* < 0.001), with Jordan reporting the lowest physical activity levels and Tunisia lower sedentary time.

**Conclusion:**

Mediterranean lifestyle adherence and its psychosocial and behavioural correlates vary substantially across countries, reflecting distinct constellations of sociocultural, socioeconomic, and lifestyle factors rather than dietary patterns alone. These findings highlight the importance of multidimensional, context-sensitive approaches to Mediterranean lifestyle promotion and provide a descriptive framework to inform tailored public-health strategies and future longitudinal and intervention research.

## 1 Introduction

Health is widely conceptualized as an integrated state of physical, mental, and social wellbeing (1). This multidimensional status does not arise randomly; it depends, to a large extent, on everyday health-related behaviours, including nutrition, sleep quality, physical activity, and the quality of social relationships and engagement (2). Accordingly, modern public-health approaches increasingly emphasize behavioural patterns rather than isolated lifestyle factors.

Within this constellation of interrelated health behaviours, dietary patterns represent a central and widely investigated component of lifestyle-related health determinants (3, 4). Rather than isolated nutrient intake, overall dietary patterns provide a more comprehensive framework for understanding how habitual eating behaviours influence health outcomes (3, 4). Among these, the Mediterranean Diet (MedDiet) is one of the most extensively investigated and consistently associated with favourable health outcomes (5). It is typically characterized by high consumption of plant-based foods, olive oil as the primary fat source, moderate intake of fish and dairy products, and limited consumption of red and processed meat and sweets (6). Substantial epidemiological and mechanistic evidence supports its protective role against cardiometabolic diseases, neurodegenerative disorders, and all-cause mortality, with proposed mechanisms including improved lipid profiles, anti-inflammatory effects, modulation of gut microbiota, and enhanced metabolic regulation (4, 5, 7, 8). Diet alone does not fully capture the historical and cultural context in which Mediterranean eating habits developed. The MedDiet has traditionally been embedded within a broader Mediterranean lifestyle that integrates regular physical activity, restorative sleep, conviviality and social engagement (3). This broader construct is operationalized through the Mediterranean Lifestyle Index (MEDLIFE index), which jointly assesses Mediterranean food consumption, Mediterranean dietary habits, and lifestyle and social behaviours (9). Growing evidence indicates that adherence to a Mediterranean lifestyle (MedLife) pattern is associated with reduced mortality and improved health outcomes beyond the effects of diet alone (3, 10), highlighting the relevance of holistic lifestyle frameworks in health research and policy.

In this perspective, MedLife adherence can be understood as a multidimensional configuration of interrelated behaviours and psychosocial conditions rather than a purely dietary pattern (3, 9). Lifestyle components such as physical activity, sleep quality, social participation, and psychological wellbeing are not only outcomes of health behaviour but may also influence the adoption and maintenance of dietary practices through behavioural, social, and environmental pathways (3, 4). Moreover, contemporary lifestyle contexts increasingly involve technology-mediated behaviours that may interact with dietary behaviour, physical activity, sedentary time, sleep, social engagement, and stress exposure (11). Considering these domains together therefore provides a more comprehensive framework for understanding how MedLife adherence manifests across populations and sociocultural contexts.

Despite its recognized benefits, adherence to the MedLife is unevenly distributed and increasingly shaped by socioeconomic, cultural, and environmental determinants (3). Structural barriers such as healthy food affordability, education level, urbanization, time constraints, and broader socioeconomic inequalities repeatedly emerged as determinants that may facilitate or hinder sustainable adoption, while psychosocial stress and sleep disturbances may further interact with lifestyle behaviours in complex bidirectional pathways (2, 3, 12–14). Global evidence showed marked geographic variability, with several studies reporting a gradual decline in adherence within Mediterranean countries themselves, while non-Mediterranean populations show increasing interest and, in some cases, higher levels of adherence (3, 6, 12, 15). Yet, even among neighboring countries that share similar historical, cultural, and socioeconomic backgrounds, systematic comparisons of MedDiet adherence and related lifestyle behaviours remain relatively scarce, with many studies focusing on single countries comparison and/or use different, non-comparable tools, which makes it difficult to draw robust conclusions about cross-country patterns and their correlates.

Within this evolving landscape, the MEDIET4ALL e-survey was established to map Mediterranean lifestyle adherence and its correlates across Mediterranean and neighboring countries using a standardized, multilingual international e-survey (16). To date, MEDIET4ALL publications have documented heterogeneity by region (15), sex (17), and determinants of adherence and health status (3, 18), and have further highlighted country-context effects in a focused Germany–Türkiye comparison (19). Collectively, these studies provide a coherent yet fragmented picture of Mediterranean lifestyle adherence, revealing variability across regions, sexes, and selected countries. However, a critical gap remains, as no MEDIET4ALL study has yet provided a comprehensive country-by-country comparison across all participating nations integrating MedLife adherence (global and domain scores) with the broader psychosocial and lifestyle domains measured in the survey. Addressing this gap is essential because aggregation can mask meaningful heterogeneity and because domain-specific profiles are needed to guide context-tailored prevention and intervention priorities.

Accordingly, the present study aims to provide a comprehensive comparative analysis of Mediterranean lifestyle adherence and associated psychosocial and lifestyle factors across 10 Mediterranean and neighboring countries participating in MEDIET4ALL. Specifically, we sought to (i) compare adherence to the Mediterranean lifestyle using the MedLife Index (global score and three domain blocks), and (ii) compare cross-country distributions of depression, anxiety, and stress; life satisfaction; sleep characteristics and insomnia severity; physical activity and sedentary behaviour; social participation; and technology use. We hypothesized that Mediterranean lifestyle adherence would differ significantly across countries. Additionally, we expected that cross-country differences in MedLife adherence would co-occur with differences in psychosocial and lifestyle factors, yielding distinct national profiles across the measured domains.

## 2 Methods

### 2.1 Survey development and participant recruitment

This cross-sectional study examined adherence to the MedDiet and MedLife, as well as potential determinant factors, using the international MEDIET4ALL electronic survey. The survey was designed, reviewed, and refined by a multidisciplinary research team (public health, nutrition, sport and movement sciences, psychology, and sociology) at Johannes Gutenberg University Mainz (Germany), in collaboration with partner institutions participating in the MEDIET4ALL PRIMA project. The overall methodology and core survey framework are consistent with those reported in prior MEDIET4ALL analyses (15, 17).

Before dissemination, the questionnaire underwent a 1-week pilot phase led by the project steering group, followed by revisions based on stakeholder feedback to improve clarity and content validity. The finalized survey was administered over a four-month period starting in summer 2024 in 10 Mediterranean and adjacent countries (Germany, France, Italy, Spain, Luxembourg, Tunisia, Algeria, Morocco, Türkiye, and Jordan).

Data were collected using the GDPR-compliant SoSci Survey platform hosted at Johannes Gutenberg University. The survey link was distributed via consortium and partner networks through email invitations, institutional and consortium webpages, and social media channels. Eligible participants were adults aged 18 years or older, with no predefined upper age limit, residing in one of the 10 participating countries and able to complete the questionnaire in one of the available survey languages. Individuals who self-reported a diagnosis of cognitive impairment were excluded from participation. Additional exclusions were applied during data screening, as described below.

Initial recruitment yielded >8,000 responses. After systematic completeness and validity screening, 4,010 responses were retained for analysis. Completeness required full survey finalization, and partial submissions were excluded. Validity checks included logic-based consistency screening (e.g., discordant physical activity responses), duplicate detection (IP address concordance, timestamp proximity, and highly similar response patterns), and removal of extreme/unrealistic values (e.g., implausible sleep durations).

The study followed the Declaration of Helsinki (20) and received ethics approval from the Ethics Committee of the Faculty of Medicine, University of Sfax (Approval Code: 058/24).

### 2.2 Data privacy and consent of participation

Participation was voluntary. No personally identifiable information (e.g., names or contact details) was collected. Participants could discontinue at any time prior to final submission; incomplete responses were automatically discarded. Submission of the completed questionnaire constituted informed consent for the anonymized scientific use of the data.

### 2.3 Survey questionnaires

The MEDIET4ALL e-survey integrates several validated questionnaires assessing Mediterranean lifestyle adherence and related behavioural, psychosocial, and health-related factors. The survey collects information on Mediterranean lifestyle adherence, psychological distress, life satisfaction, sleep characteristics, physical activity and sedentary behaviour, social participation, technology use, and key sociodemographic variables. Detailed descriptions of the MEDIET4ALL survey framework, questionnaire items, and methodological procedures have been reported in previous publications from the project (1, 15–19). The instruments included in the survey have been widely used in international research and have demonstrated acceptable psychometric properties across diverse cultural contexts. The survey was available in seven languages (English, German, French, Italian, Spanish, Arabic, and Turkish). Items without existing official translations were translated and back-translated using standard procedures (21), and all language versions demonstrated excellent test–retest reliability (*r* = 0.81–0.94). This harmonized measurement approach was intended to maximize cross-country comparability and continuity with previous MEDIET4ALL analyses.

#### 2.3.1 Life satisfaction (SLSQ)

Life satisfaction was assessed using the Short Life Satisfaction Questionnaire (SLSQ), a brief adaptation derived from the Satisfaction with Life Scale framework (22) and previously applied in large-scale lockdown research (23). The SLSQ includes three items rated on a 7-point Likert scale (1 = strongly disagree to 7 = strongly agree), yielding a total score from 3 to 21 (higher scores reflect higher life satisfaction).

#### 2.3.2 Psychological distress (DASS-21)

Depression, anxiety, and stress symptoms over the preceding week were evaluated using the Depression Anxiety Stress Scales-21 (24). The 21 items form three subscales (7 items each), rated from 0 (“did not apply to me”) to 3 (“applied to me very much/most of the time”). Subscale totals were multiplied by two to align with DASS-42 scoring guidelines (24).

#### 2.3.3 Sleep quantity, quality, latency, and efficiency

A shortened set of items derived from the Pittsburgh Sleep Quality Index (25) assessed four sleep dimensions over the previous month including sleep efficiency (calculated as total sleep time divided by time spent in bed and categorized as >85% vs. ≤85%), sleep latency (<20 min vs. ≥20 min), subjective sleep quality (rated on a four-point scale from very good to very bad), and sleep duration classified according to age-specific recommendations (26).

#### 2.3.4 Technology use (STuQL)

Technology use for social participation, dietary practices, and physical activity was assessed using the Short Technology-Use Questionnaire (STuQL), previously used in confinement-related lifestyle research (27). It contains three items rated on a 5-point scale (never to all the time), with total scores ranging from 3 to 15.

#### 2.3.5 Insomnia severity (ISI)

Insomnia symptom severity and impact were measured with the ISI (24). The 7 items are scored from 0 to 4, producing a total score from 0 to 28, with established severity thresholds (28).

#### 2.3.6 Social participation (SSPQ)

Social participation over the preceding 12 months was assessed using the Short Social Participation Questionnaire (SSPQ), previously validated and applied in confinement-related studies (23). The instrument includes 14 items (10 Likert-type items and 4 yes/no items), summed to a total score from 14 to 70 (higher scores indicate greater participation).

#### 2.3.7 Physical activity (IPAQ-SF)

Physical activity was assessed using the IPAQ-SF (29). The IPAQ-SF estimates weekly activity (MET-min/week) across vigorous, moderate, and walking activities and supports classification into low (<1,500), moderate (1,500–2,999), and high (≥3,000) activity categories (29). Mediterranean lifestyle adherence (MEDLIFE Index).

MedLife adherence was evaluated using the MEDLIFE Index (9), selected based on prior evidence supporting its conceptual and psychometric utility among existing MedDiet/MedLife indices (30). The MEDLIFE Index comprises 28 dichotomous items (0 = non-adherence; 1 = adherence), grouped into three domains (blocks): (i) food consumption frequency (15 items), (ii) MedDiet habits (7 items), and (iii) lifestyle behaviours (6 items). In addition to the overall MEDLIFE score, domain-specific (block) scores were calculated to examine cross-country differences across these three dimensions of the Mediterranean lifestyle. Total scores range from 0 to 28 and were categorized into low (<12), medium (12–14, 31, 32), and high (>16) adherence using tertile-derived cut-points (3).

### 2.4 Statistical analyses

All statistical analyses were performed using Statistica (version 12). Descriptive data are presented as mean ± standard deviation (SD). The distribution of continuous variables was evaluated using the Shapiro–Wilk test. Because assumptions of normality were not consistently met, between-country differences were examined using the Kruskal–Wallis *H* test (33). When a significant country effect was detected, pairwise comparisons were conducted using Dunn’s *post hoc* test with adjustment for multiple testing (34). The effect size for the main (country) effect was calculated as eta-squared (η^2^), interpreted using conventional benchmarks: small (≈0.01), medium (≈0.06), and large (≈0.14) (35). Statistical significance was set at *p* < 0.05.

The MedLife Index was defined as the primary outcome, as it represents a composite indicator integrating dietary, lifestyle, and social dimensions of the Mediterranean lifestyle. Psychosocial indicators and behavioural variables were analysed as secondary outcomes to provide complementary information on multidimensional lifestyle profiles across countries.

## 3 Results

### 3.1 Medlife Index

**Figure 1:**
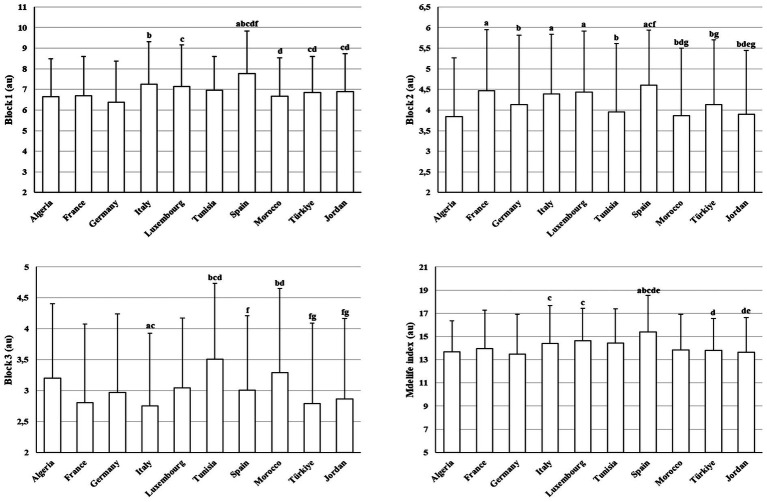
Between-country differences in MEDLIFE block scores (mean ± SD). a: Significantly different compared to Algeria; b: significantly different compared to France; c: significantly different compared to Germany; d: significantly different compared to Italy; e: significantly different compared to Luxembourg; f: significantly different compared to Tunisia; g: significantly different compared to Spain; h: significantly different compared to Morocco; i: significantly different compared to Türkiye.

Figure 1 presents between-country differences in MEDLIFE block and total scores. Block 1 differed significantly between countries (H(9) = 120.84, *p* < 0.001, *η*^2^ = 0.07). Spain exhibited higher scores than several countries, including Algeria, France, Germany, Tunisia, Morocco, Türkiye, and Jordan (*z* = 3.29–9.03, adjusted *p* < 0.001–0.005). Conversely, Germany and France showed consistently lower Block 1 scores than Spain and Italy (*z* = 3.29–9.03, adjusted *p* < 0.001–0.005).

Block 2 also differed significantly between countries (H(9) = 95.94, *p* < 0.001, *η*^2^ = 0.09). Algeria showed lower scores compared with France, Italy, Luxembourg, and Spain (*z* = 3.34–6.50, adjusted *p* < 0.001–0.05). Spain and France frequently ranked among higher-scoring countries relative to Morocco, Türkiye, and Jordan (*z* = 3.34–6.50, adjusted *p* < 0.001–0.05).

Block 3 showed significant cross-country variation (H(9) = 78.84, *p* < 0.001, *η*^2^ = 0.11). Tunisia and Morocco tended to score higher than several European countries, including France and Italy (*z* = 3.26–6.50, adjusted *p* < 0.001–0.05). Italy and France showed lower scores relative to North African countries (*z* = 3.92–6.50, adjusted *p* < 0.001–0.05).

Global MedLife Index scores differed significantly between countries (H(9) = 103.99, *p* < 0.001, *η*^2^ = 0.08). Spain displayed consistently higher global scores than Algeria, France, Germany, Tunisia, Morocco, Türkiye, and Jordan (*z* = 3.42–8.12, adjusted *p* < 0.001–0.02). Germany scored lower than Italy and Luxembourg (*z* = 3.62–8.12, adjusted *p* < 0.001–0.05), while Italy scored higher than Türkiye and Jordan (*z* = 3.42–4.62, adjusted *p* < 0.001–0.02).

**Figure 2:**
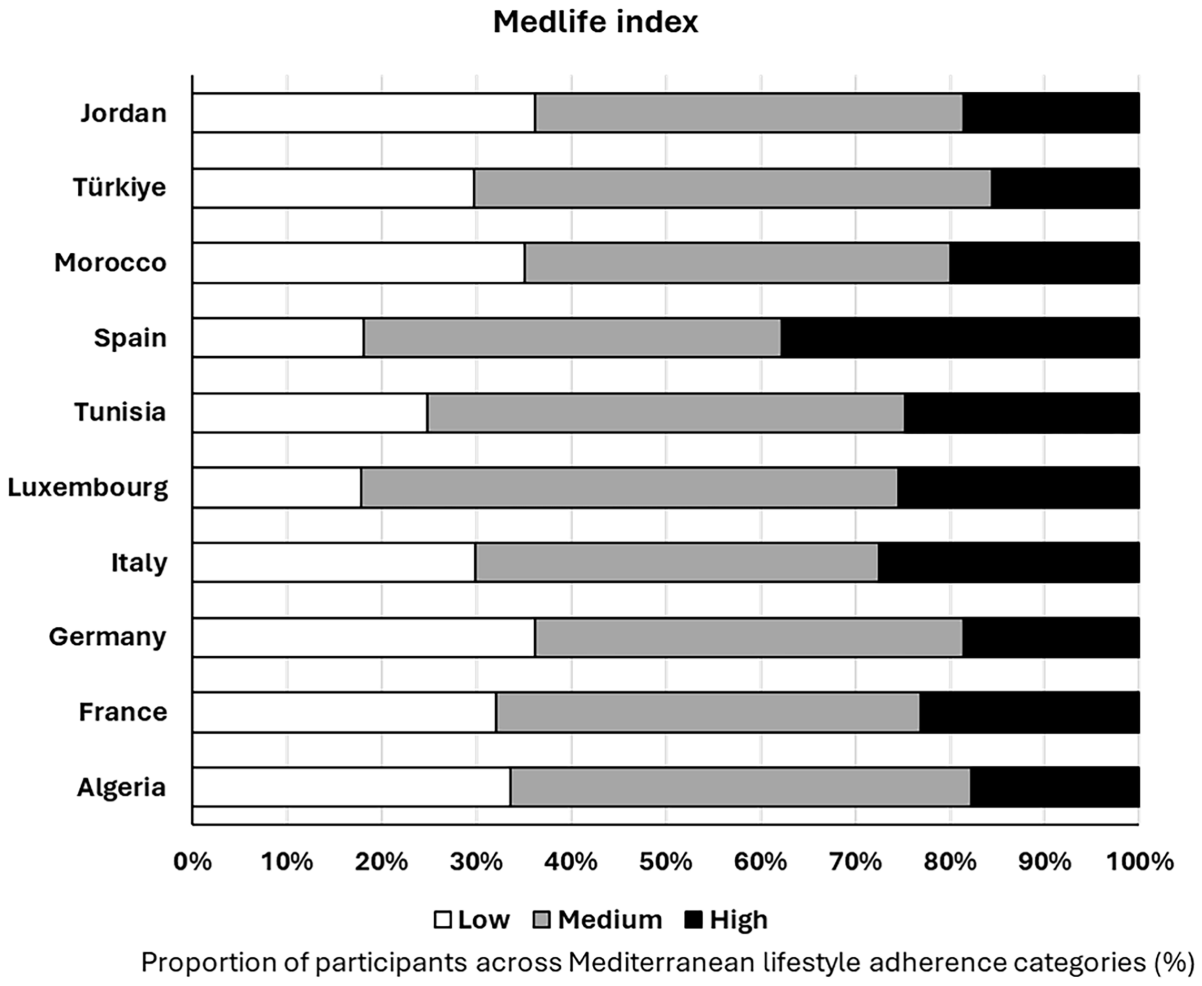
Medlife Index group levels (%) by country.

Figure 2 illustrates the distribution of Mediterranean lifestyle adherence categories across countries. The distribution of MedLife adherence levels differed significantly between countries (*χ*^2^ = 113:936, *p* < 0.001), with Spain and Italy showing relatively higher proportions of participants in the high-adherence category, whereas lower proportions were observed in several non-Mediterranean and North African contexts. Conversely, the prevalence of low adherence tended to be more pronounced in Jordan, Morocco, and Germany.

### 3.2 Psychological distress and life satisfaction

**Figure 3:**
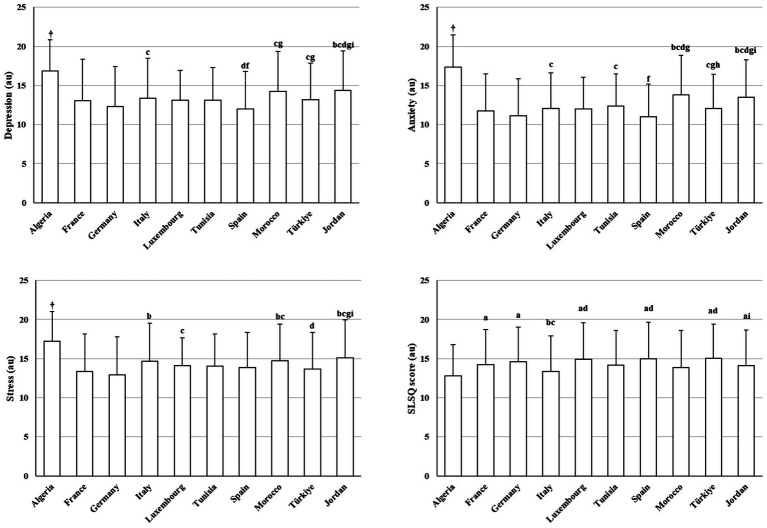
Between-country differences in DASS-21 and life satisfaction scores (mean ± SD). ^†^Significantly different compared to all countries; a: significantly different compared to Algeria; b: significantly different compared to France; c: significantly different compared to Germany; d: significantly different compared to Italy; f: significantly different compared to Tunisia; g: significantly different compared to Spain; h: significantly different compared to Morocco; i: significantly different compared to Türkiye.

Figure 3 presents between-country differences in psychological distress (DASS-21) and life satisfaction scores. Country effects were significant for depression (H(9) = 189.02, *p* < 0.001, *η*^2^ = 0.04), anxiety (H(9) = 312.82, *p* < 0.001, *η*^2^ = 0.02), and stress (H(9) = 177.97, *p* < 0.001, *η*^2^ = 0.05). Algeria exhibited markedly higher scores across all three domains relative to other countries (*z* = 3.85–14.29, adjusted *p* < 0.001–0.05). Germany generally showed lower anxiety and stress compared with several countries (*z* = 3.32–10.63, adjusted *p* < 0.001–0.03), whereas Jordan frequently ranked among higher-distress profiles.

Similarly, life satisfaction differed significantly between countries (H(9) = 81.62, *p* < 0.001, *η*^2^ = 0.02). Algeria reported lower life satisfaction relative to several European countries (e.g., France, Germany, Luxembourg, Spain, and Türkiye), while Italy tended to show lower scores compared with Germany, Luxembourg, Spain, and Türkiye (*z* = 3.34–6.34, adjusted *p* < 0.001–0.05).

The distribution of categorical outcomes across countries is additionally illustrated in Supplementary Figure S1.

### 3.3 Social participation, physical activity and sitting time

**Figure 4:**
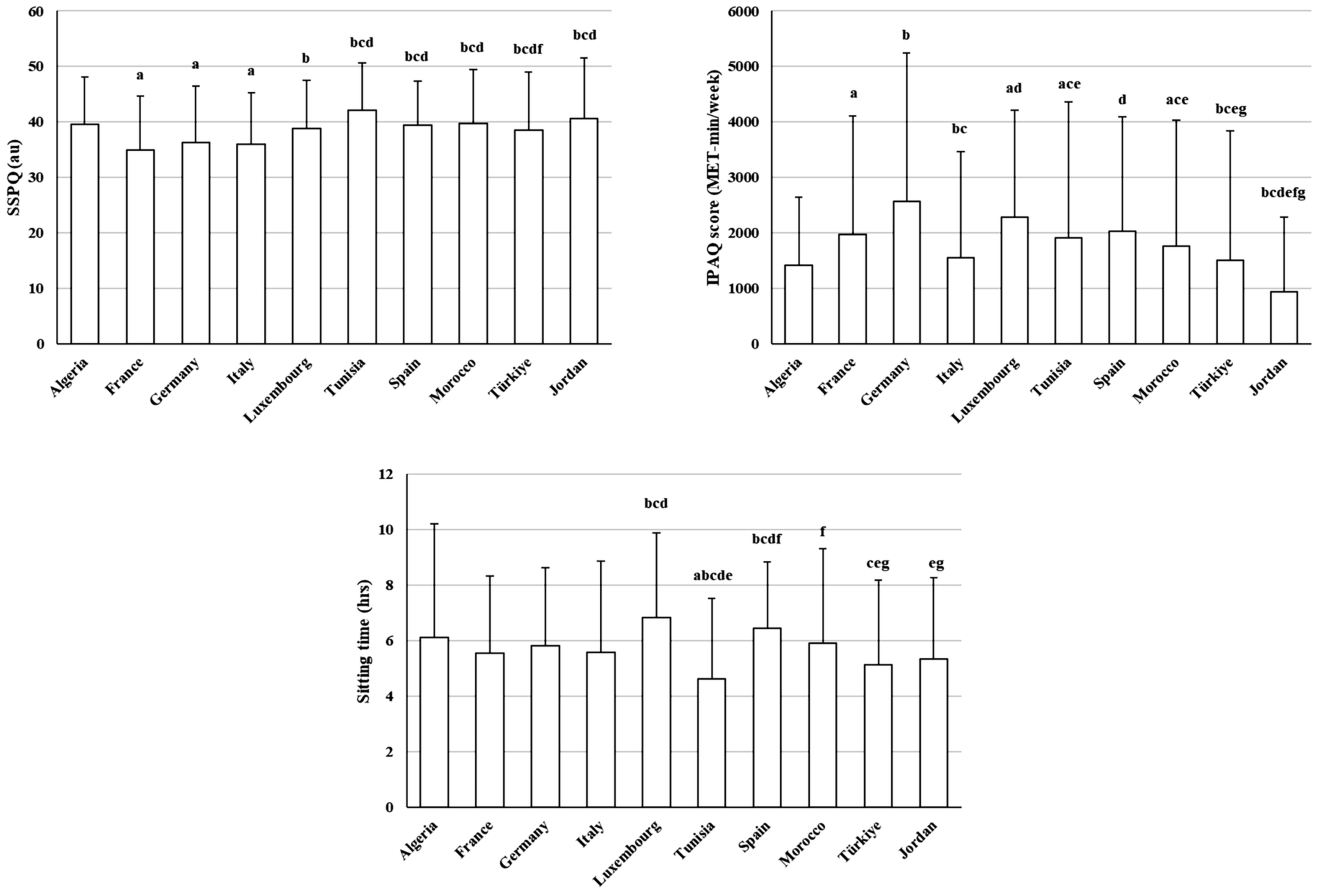
Between-country differences in social participation score, IPAQ score, and sitting time (mean ± SD). a: Significantly different compared to Algeria; b: Significantly different compared to France; c: Significantly different compared to Germany; d: Significantly different compared to Italy; e: Significantly different compared to Luxembourg; f: Significantly different compared to Tunisia; g: Significantly different compared to Spain; i: Significantly different compared to Türkiye.

Figure 4 presents between-country differences in social participation scores, physical activity (IPAQ) scores, and sitting time. Social participation score varied substantially across countries (H(9) = 194.86, *p* < 0.001, *η*^2^ = 0.04). France, Germany, and Italy showed lower SSPQ scores than Tunisia, Spain, Morocco, Türkiye, and Jordan (*z* = 3.79–9.31, adjusted *p* < 0.001–0.05). Conversely, Tunisia and Morocco demonstrated higher social participation levels compared with several European countries (*z* = 3.79–8.13, adjusted *p* < 0.001–0.05).

Physical activity differed strongly between countries (H(9) = 357.29, *p* < 0.001, *η*^2^ = 0.02), with Jordan showing consistently lower IPAQ score than all other countries, except Algeria, (*z* = 3.57–14.72, adjusted *p* < 0.001–0.05), while Germany followed by Luxembourg and Spain generally exhibited higher physical activity levels compared with Italy, Tunisia, Morocco, and Türkiye (*z* = 3.57–14.72, adjusted *p* < 0.001–0.05). Sitting time also differed significantly (H(9) = 103.31, *p* < 0.001, *η*^2^ = 0.08), with Luxembourg and Spain often showing higher sitting time than several countries, whereas Tunisia tended to report lower sitting time than most countries (*z* = 3.32–7.21, adjusted *p* < 0.001–0.05).

The distribution of categorical outcomes across countries is additionally illustrated in Supplementary Figure S2.

### 3.4 Sleep, insomnia, and technology use behaviour

**Figure 5:**
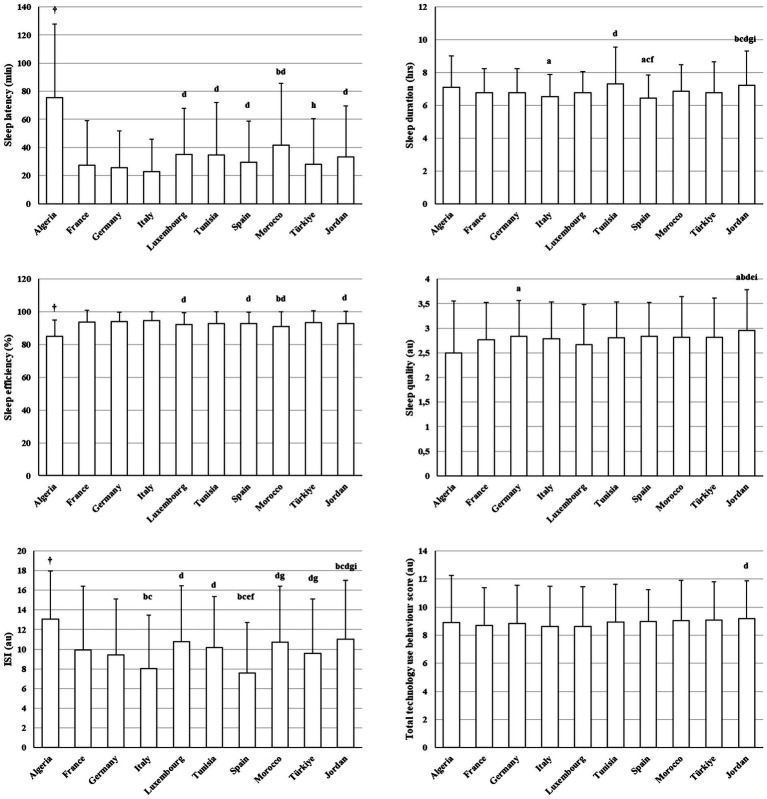
Between-country differences in sleep parameters, insomnia, and technology use behaviours (mean ± SD). ^†^Significantly different compared to all countries; a: Significantly different compared to Algeria; b: Significantly different compared to France; c: Significantly different compared to Germany; d: Significantly different compared to Italy; e: Significantly different compared to Luxembourg; f: Significantly different compared to Tunisia; g: Significantly different compared to Spain; h: Significantly different compared to Morocco; i: Significantly different compared to Türkiye.

Figure 5 presents between-country differences in sleep patterns, Insomnia Severity Index (ISI) scores, and technology use behaviours.

Sleep indicators differed significantly between countries (sleep latency: H(9) = 198.63, *p* < 0.001, *η*^2^ = 0.04; sleep duration: H(9) = 66.64, *p* < 0.001, *η*^2^ = 0.13; sleep efficiency: H(9) = 166.07, *p* < 0.001, *η*^2^ = 0.05; subjective sleep quality: H(9) = 49.99, *p* < 0.001, *η*^2^ = 0.05). Algeria consistently exhibited a less favourable sleep profile, characterized by higher sleep latency and poorer sleep efficiency compared with all other countries (*z* = 3.40–12.82, adjusted *p* < 0.001–0.05), and lower subjective sleep quality relative to Germany. In contrast, Jordan tended to show a more favourable sleep profile, with longer sleep duration and higher subjective sleep quality than several European countries (*z* = 3.36–6.05, adjusted *p* < 0.001–0.05). France and Morocco also exhibited relatively higher sleep latency compared with multiple countries (*z* = 3.45–3.46, adjusted *p* = 0.02), whereas Italy demonstrated lower latency and relatively better sleep efficiency compared with Luxembourg, Tunisia, Spain, Morocco, and Jordan (*z* = 3.40–5.14, adjusted *p* < 0.001–0.05).

Insomnia severity differed significantly across countries (H(9) = 192.15, *p* < 0.001, *η*^2^ = 0.04). Algeria reported higher ISI scores than all other countries (*z* = 3.34–9.77, adjusted *p* < 0.001–0.05). France and Germany also exhibited higher insomnia severity compared with Italy and Spain (*z* = 4.20–5.18, adjusted *p* < 0.001), whereas Jordan tended to show higher ISI scores than several European countries (*z* = 3.39–4.69, adjusted *p* < 0.001–0.03).

Significant country differences were observed for Technology use behaviours score (H(9) = 17.56, *p* = 0.041, *η*^2^ = 0.01). Italy reported lower technology use behaviour compared to Jordan (*z* = 3.28, *p* < 0.047), while other countries showed broadly comparable levels.

## 4 Discussion

The present MEDIET4ALL analysis provides a comprehensive country-by-country comparison of Mediterranean lifestyle adherence and a broad set of co-occurring psychosocial and lifestyle correlates across 10 Mediterranean and neighboring countries. Several consistent patterns emerged. First, Mediterranean lifestyle adherence differed markedly between countries at both global and domain levels, with Spain showing one of the most favourable MedLife profiles, whereas several non-Mediterranean and North African contexts exhibited comparatively lower adherence. Second, pronounced heterogeneity was observed in psychological distress and insomnia severity, with a distinct high-burden profile in Algeria and, more broadly, higher distress and sleep-related problems in North African and Middle Eastern contexts compared with several European countries. Third, meaningful cross-country differences were found in life satisfaction and social participation, with particularly large contrasts in social participation and generally lower levels in some European contexts relative to North African and Middle Eastern countries (e.g., Tunisia, Morocco and Jordan). Fourth, physical activity and sedentary behaviour varied substantially across countries, with Germany reporting higher physical activity levels and Tunisia showing lower sedentary time. Finally, sleep parameters differed markedly across countries, with especially large contrasts in sleep latency, efficiency, and ISI with comparatively less favourable sleep indicators and higher ISI observed in some North African (e.g., Algeria and Morocco) context and more favourable indicators in others (e.g., Spain and Italy).

Collectively, these findings reinforce that MedLifestyle adherence is context-dependent and that similar global adherence levels may conceal distinct country-specific behavioural and psychosocial profiles. This interpretation extends earlier MEDIET4ALL evidence of heterogeneity by region (15), sex (17), and selected country comparisons (19). Importantly, the present study addresses a remaining gap by moving beyond aggregated comparisons and single-country analyses to provide a granular, multi-domain profiling of 10 countries, thereby offering a deeper and more detailed empirical basis for context-sensitive prevention strategies and policy interventions.

### 4.1 MedLife Index: country-level heterogeneity and domain-specific patterns

The MedLife Index differed significantly between countries, and the presence of significant effects across Blocks 1–3 indicates that national differences were not confined to a single behavioural domain. This is relevant because the Mediterranean lifestyle is inherently multidimensional (3). Global scores can conceal “compensatory patterns,” whereby higher dietary adherence co-exists with poorer sleep or lower social participation, or vice versa. A similar phenomenon has been observed in sex-specific MEDIET4ALL analyses, which revealed domain-level differences between men and women despite comparable global adherence (17). Extending this concept to the country level, the present findings suggest that interventions should be oriented toward country-specific domain deficits rather than treating Mediterranean lifestyle adherence as a uniform behavioural construct.

Beyond domain-specific differences, the results indicate that Mediterranean countries tended, on average, to display more favourable dietary-related patterns, albeit with substantial heterogeneity, reinforcing the notion that geographic proximity does not necessarily translate into behavioural homogeneity. In the present analysis, Spain consistently emerged among the countries with higher global MedLife scores relative to multiple contexts (e.g., Algeria, France, Germany, Tunisia), whereas Germany and France exhibited lower global adherence compared with Spain and/or Italy and Luxembourg. These patterns are broadly consistent with previous regional MEDIET4ALL findings (15), but they also demonstrate that a simple “Mediterranean vs non-Mediterranean” categorization is insufficient, as meaningful variability exists within both Mediterranean and neighboring regions.

Beyond continuous scores, the distribution of MedLife adherence categories further illustrated cross-country heterogeneity. Spain and Italy showed relatively higher proportions of participants in the high-adherence category, whereas several other contexts exhibited larger proportions of low or moderate adherence, including countries such as Jordan, Morocco, and Germany. This categorical perspective complements the continuous-score analysis and supports the notion that Mediterranean lifestyle patterns vary not only in degree but also in their population-level distribution across countries.

Such heterogeneity aligns with broader international evidence indicating that adherence to the Mediterranean lifestyle varies widely across populations and is shaped by sociocultural and economic contexts (4, 6, 12). Several studies have reported a gradual decline in adherence within parts of the Mediterranean region alongside increasing interest in Mediterranean-like dietary patterns in some non-Mediterranean populations (6, 12, 36), suggesting that modernization, urbanization, and evolving food environments may progressively reshape traditional lifestyle patterns (3, 4, 37).

From an interpretive perspective, cross-country differences in MedLife adherence may reflect variations in demographic composition, socioeconomic conditions, psychosocial profiles, and lifestyle behaviours (3, 12–15, 38). Evidence from the MEDIET4ALL determinants analyses indicates that higher Mediterranean lifestyle adherence is associated with higher educational attainment, greater awareness of MedLife principles, lower perceived barriers, normal BMI, better self-reported health status, and more favourable psychosocial profiles (3, 19). Moreover, individuals with higher adherence tend to report greater social participation, higher physical activity levels, better sleep quality, and lower psychological distress, highlighting the clustering of behavioural and psychosocial factors within lifestyle profiles.

In this context, the relatively favourable MedLife profile observed in Spain may reflect the coexistence of several facilitating conditions identified within the MEDIET4ALL framework. In the present analysis, Spain consistently ranked among the countries with higher global MedLife scores and showed comparatively favourable patterns in dietary adherence, social participation, and physical activity, alongside relatively balanced psychosocial and sleep-related indicators. By contrast, several North African and Middle Eastern countries displayed less favourable MedLife profiles, often characterized by higher psychological distress, greater sleep disturbances, or lower physical activity levels. These patterns mirror determinants and barriers previously reported within the MEDIET4ALL population, including higher psychosocial burden, perceived structural constraints, and less supportive lifestyle environments (3, 13). Similarly, some Central and Western European countries, despite relatively favourable socioeconomic conditions, exhibited lower social participation or domain-specific deficits, which may partly explain their comparatively lower global MedLife scores. This observation resonates with earlier MEDIET4ALL findings showing that lifestyle domains do not necessarily evolve uniformly across contexts and that high socioeconomic status does not automatically translate into holistic Mediterranean lifestyle adherence.

Taken together, these cross-country contrasts suggest that variations in MedLife adherence are not driven by dietary factors alone but rather reflect distinct constellations of behavioural, psychosocial, and contextual characteristics. In this sense, the present findings extend previous MEDIET4ALL analyses by illustrating how different combinations of facilitators and constraints may shape country-specific Mediterranean lifestyle profiles, thereby contributing to the observed heterogeneity in both global and domain-specific adherence patterns. Importantly, Block-specific differences further suggest that dietary habits, contextual lifestyle practices, and social-lifestyle components do not evolve uniformly across countries. This reinforces the notion that Mediterranean lifestyle adherence cannot be reduced to dietary behaviour alone and highlights the need for domain-targeted, culturally sensitive intervention strategies rather than purely diet-centered approaches.

### 4.2 Psychosocial wellbeing: cross-country gradients in psychological distress and life satisfaction

The present findings show substantial cross-country variability in psychosocial wellbeing, captured by both psychological distress (depression, anxiety, stress) and life satisfaction. Several countries exhibited comparatively elevated distress across multiple DASS-21 domains, whereas others showed more favourable psychosocial profiles. For example, some European contexts (notably Germany and Spain) tended to report lower levels of depressive and anxiety symptoms than several other countries in multiple post-hoc contrasts, while higher distress profiles were more frequently observed in some non-European contexts (e.g., Jordan and Morocco) across anxiety and/or depression comparisons. In parallel, life satisfaction also differed across countries, with higher satisfaction levels in several European settings (e.g., Germany, Luxembourg, Spain), and lower satisfaction observed in some other contexts (e.g., Jordan compared with Türkiye).

Although causal inference is not possible in this cross-sectional survey, the convergence of negative (distress) and positive (life satisfaction) indicators suggests that psychosocial wellbeing tends to cluster with broader lifestyle profiles (39–41). Importantly, the present dataset includes several lifestyle correlates that plausibly co-vary with psychosocial status. For instance, contexts characterized by higher psychological burden also tended to show less favourable sleep-related outcomes and/or insomnia severity (e.g., Jordan showing higher insomnia severity and sleep latency than some European contexts). Likewise, large cross-country differences in physical activity may be relevant for interpretation. Indeed, Jordan consistently reported the lowest IPAQ levels compared with all other countries, a pattern that may align with a less favourable psychosocial profile in some settings, given well-established links between low activity and poorer mental health (42, 43).

These co-occurring patterns support an integrated interpretation in which psychological distress, sleep disturbance, and movement behaviours jointly contribute to country-specific psychosocial–lifestyle configurations.

Insights from the MEDIET4ALL framework provide a coherent interpretive lens for these gradients. Previous regional analyses documented geographic variability in psychosocial factors and perceived barriers (15), and determinant models highlighted associations between lifestyle behaviours, sociodemographic characteristics, and psychological status (3, 19). Within this framework, the current findings suggest that psychological distress and life satisfaction represent complementary components of context-dependent lifestyle profiles rather than isolated outcomes.

From a public-health perspective, the observed cross-country gradients in distress and life satisfaction reinforce that Mediterranean lifestyle promotion should not be framed as diet-only messaging. In settings characterized by elevated psychological burden and poorer sleep outcomes, interventions may need to integrate mental-health-sensitive components (e.g., stress management, sleep hygiene, and support for sustainable physical activity) alongside dietary guidance to better match the multidimensional nature of Mediterranean lifestyle patterns and the country-specific needs highlighted by this comparative analysis.

### 4.3 Social participation: cross-country variability within Mediterranean lifestyle profiles

Social participation showed pronounced cross-country differences, with several European contexts (notably France, Germany, and Italy) reporting lower SSPQ scores relative to multiple other countries. In contrast, higher levels of social participation were observed in several Mediterranean and neighboring contexts, including Tunisia, Spain, Morocco, Türkiye, and Jordan. This pattern highlights substantial heterogeneity in the social dimension of lifestyle behaviours across countries.

This finding is particularly relevant because conviviality and social connectedness constitute core dimensions of the Mediterranean lifestyle, potentially shaping dietary habits, physical activity patterns, and psychosocial wellbeing through shared practices, collective meals, and social norms (3, 44, 45). The present results extend previous MEDIET4ALL evidence, including the Germany–Türkiye comparison, which identified social participation as a key differentiating domain between national contexts (19). The current multi-country analysis further indicates that social participation represents one of the most variable lifestyle dimensions across countries, suggesting that national contexts differ not only in dietary adherence but also in the social environments that support or constrain Mediterranean lifestyle behaviours.

More broadly, the observed cross-cultural variability in social participation may reflect differences in family structures, community engagement traditions, urbanization patterns, and social norms across countries (3, 15, 46). For example, contexts characterized by stronger family networks and community-oriented social practices tended to show higher levels of social participation, whereas more urbanized and individual-oriented contexts tended to display lower levels. Such patterns are consistent with MEDIET4ALL findings and broader international evidence identifying sociocultural and structural factors as important correlates of lifestyle behaviours and wellbeing (13–15).

Importantly, interpretation must remain cautious, as cross-cultural differences in self-reported social participation may also be influenced by cultural norms, social desirability, and measurement equivalence. Nevertheless, the magnitude and consistency of cross-country differences suggest that Mediterranean lifestyle promotion should explicitly consider the social participation environment, including community structures, family networks, and opportunities for collective engagement, rather than focusing solely on individual behavioural change.

### 4.4 Physical activity and sedentary behaviour: distinct but interrelated behavioural domains

Physical activity and sedentary behaviour exhibited marked cross-country differences, highlighting their relevance as distinct yet interrelated components of Mediterranean lifestyle profiles. In the present analysis, Jordan consistently reported the lowest physical activity levels compared with all other countries, indicating that physical activity may represent a key limiting domain within certain national contexts. At the same time, sedentary behaviour varied substantially across countries, with Luxembourg and Spain frequently showing higher sitting time, whereas Tunisia reported comparatively lower sedentary behaviour in multiple contrasts.

More broadly, several European contexts (e.g., Germany, Luxembourg, and Spain) tended to display higher physical activity levels, whereas Tunisia exhibited lower sedentary behaviour, illustrating that physical activity and sitting time do not necessarily follow the same cross-country gradient. These findings highlight the importance of treating physical activity and sedentary behaviour as related but distinct behavioural targets, as countries may exhibit favourable profiles in one domain but not in the other.

The observed cross-country patterns are consistent with MEDIET4ALL determinant analyses and international evidence indicating that physical activity and sedentary behaviour are shaped by contextual factors such as urban design, occupational demands, cultural norms, and access to recreational opportunities (3, 14). Within the present dataset, movement behaviours also tended to align with psychosocial and sleep-related profiles, reinforcing the concept of clustered lifestyle patterns across countries.

From a public-health perspective, these findings suggest that Mediterranean lifestyle promotion should incorporate context-sensitive strategies addressing both physical activity and sedentary behaviour, particularly given their consistent associations with Mediterranean lifestyle adherence, psychosocial wellbeing, and self-reported health status observed within the MEDIET4ALL framework (3, 19) and other population-based studies (47–49). In settings characterized by low activity levels, interventions may prioritize opportunities for structured and incidental movement, whereas contexts with high sedentary time may require targeted approaches aimed at reducing prolonged sitting in daily routines.

### 4.5 Sleep patterns and insomnia severity: cross-country variability within lifestyle profiles

Sleep parameters and insomnia severity differed significantly across countries, highlighting substantial cross-national variability in sleep-related health. Several contexts displayed less favourable sleep profiles, characterized by higher insomnia severity and longer sleep latency, whereas others exhibited comparatively more favourable sleep patterns. More specifically, less favourable sleep profiles were observed in several North African and Middle Eastern contexts, where higher insomnia severity and longer sleep latency were reported compared with multiple European countries. For instance, Jordan and Morocco tended to show higher insomnia severity and longer sleep latency than several European contexts, whereas Spain and Italy generally exhibited more favourable sleep indicators. Similarly, France and Germany displayed higher insomnia severity compared with Italy and Spain in multiple contrasts, highlighting that sleep disturbances are not restricted to non-European settings but also vary within Europe.

Conversely, more favourable sleep patterns were observed in some Southern European contexts, particularly Spain and Italy, which tended to report lower insomnia severity and shorter sleep latency relative to several other countries. These patterns suggest that sleep health may represent a critical but unevenly distributed dimension of Mediterranean lifestyle profiles across countries.

These cross-country patterns align with MEDIET4ALL determinant analyses, which identified sleep quality as an important correlate of Mediterranean lifestyle adherence and perceived health status (3, 19). Beyond cross-country contrasts, the observed sleep variability resonates with broader evidence indicating that sleep behaviours are shaped by sociocultural, occupational, and psychosocial factors, including stress, work schedules, and urban environments (50, 51). Within the present dataset, sleep profiles also tended to co-occur with psychological distress patterns, reinforcing the notion that sleep and mental health constitute interconnected dimensions within broader lifestyle configurations.

Taken together, these findings suggest that sleep should be considered an integral component of Mediterranean lifestyle interventions, particularly in contexts where sleep disturbances and psychological burden co-occur. Rather than treating sleep as an ancillary outcome, future public-health strategies may benefit from integrating sleep-health promotion alongside dietary and physical activity interventions within a multidimensional lifestyle framework.

### 4.6 Technology use: emerging behavioural dimension within lifestyle profiles

Technology use behaviour differed significantly across countries, although the magnitude of differences was smaller than that observed for other lifestyle domains. Despite the modest effect size, technology-related behaviours remain relevant given their growing influence on sleep patterns, sedentary behaviour, and psychosocial wellbeing (52–54).

Cross-country differences in technology use were less pronounced than differences in dietary, social, or psychosocial domains, suggesting that technology-related behaviours may be less culturally differentiated within the MEDIET4ALL sample. Nevertheless, international evidence indicates that digital behaviours can interact with broader lifestyle patterns, particularly through their associations with screen time, sleep quality, physical inactivity, and mental health outcomes (55–58). Importantly, the health relevance of technology use may depend less on the frequency of digital engagement itself and more on how and when technology is integrated into daily routines, including its timing, purpose, and relationship with sleep, physical activity, and social interaction (11, 31, 57–59). In this sense, technology-related behaviours may function less as a standalone lifestyle domain and more as a contextual behavioural layer that can either support or undermine healthy lifestyle practices (11, 58, 59).

Taken together, these findings suggest that technology use represents an emerging but context-dependent dimension of Mediterranean lifestyle profiles, warranting consideration in future lifestyle research and intervention strategies, even when cross-country variability appears relatively modest. Future MedLife promotion strategies may therefore benefit from considering digital environments not only as potential risk factors (e.g., excessive screen exposure and sedentary behaviour) but also as possible channels for delivering culturally adapted health promotion and lifestyle-support interventions.

### 4.7 Strengths and limitations

This study has several strengths that should be interpreted in light of limitations commonly reported in MEDIET4ALL analyses and comparable international survey-based investigations. A key strength is the large multinational sample and the inclusion of 10 Mediterranean and neighboring countries assessed using a standardized, multilingual survey and harmonized measurement framework. The simultaneous assessment of multiple lifestyle domains, including diet, physical activity, sleep, psychosocial status, social participation, and technology use, enabled a comprehensive and multidimensional characterization of Mediterranean lifestyle profiles across countries. The use of validated instruments and consistent analytic procedures further enhances the comparability and interpretability of cross-country findings. In addition, the present country-by-country approach extends previous MEDIET4ALL studies that focused on regional, sex-specific, determinant-based, or bilateral comparisons, thereby providing a more granular descriptive framework for understanding cross-national heterogeneity.

Several limitations should also be acknowledged. First, the cross-sectional design precludes causal inference, and observed associations should be interpreted as correlational rather than directional. Second, reliance on self-reported measures may introduce recall bias and social desirability bias, particularly for lifestyle behaviours such as physical activity, diet, and sleep. Third, online recruitment may have resulted in selection bias, with potential overrepresentation of individuals with higher education or digital literacy. Fourth, the samples constitute convenience samples and are not fully representative of the respective national populations. Sample composition also differed between countries, including variations in sociodemographic characteristics, which may influence the constructs examined and limit strict cross-country comparability and generalizability of country-specific findings. Fifth, sample sizes varied across countries, which may further influence the stability of some estimates. Sixth, although validated instruments were used, cross-cultural differences in interpretation and response styles may affect measurement equivalence across contexts. Finally, multiple testing in cross-country comparisons increases the risk of type I error, although post-hoc corrections were applied.

Taken together, these strengths and limitations indicate that the present findings provide a comprehensive descriptive overview of cross-country Mediterranean lifestyle patterns while highlighting the need for cautious interpretation and complementary longitudinal and experimental research designs.

## 5 Conclusions and public-health implications

This MEDIET4ALL country-by-country analysis reveals substantial heterogeneity in Mediterranean lifestyle adherence and its psychosocial and behavioural correlates across 10 Mediterranean and neighboring countries. Differences were observed not only in global MedLife adherence but also across specific lifestyle domains, including dietary patterns, physical activity, sleep, social participation, psychological distress, and technology use. These findings suggest that Mediterranean lifestyle adherence is multidimensional and context-dependent, and that similar global adherence levels may conceal distinct national configurations of behavioural and psychosocial factors. Importantly, these differences were evident not only in mean MedLife scores but also in the population-level distribution of adherence categories, indicating that countries differ both in overall adherence levels and in the proportion of individuals with high or low Mediterranean lifestyle adherence.

Countries characterized by more favourable MedLife profiles tended to display more balanced combinations of dietary adherence, physical activity, social participation, and psychosocial wellbeing, whereas less favourable profiles were often accompanied by higher psychological burden, sleep disturbances, or lower engagement in key lifestyle domains. These cross-country differences suggest that Mediterranean lifestyle patterns are shaped by complex constellations of sociocultural, socioeconomic, and behavioural characteristics rather than by dietary factors alone. This interpretation extends previous MEDIET4ALL determinants-based analyses by illustrating how different combinations of facilitators and constraints may contribute to country-specific lifestyle profiles.

From a public-health perspective, the present findings highlight the need for context-sensitive and domain-targeted intervention strategies rather than uniform Mediterranean diet promotion. Interventions aiming to foster Mediterranean lifestyle adoption may benefit from simultaneously addressing diet, movement behaviours, sleep health, psychosocial wellbeing, and social participation, with priorities adapted to country-specific deficits and strengths. For example, in countries showing comparatively lower MedLife adherence and/or lower physical activity levels, such as Jordan, interventions may prioritize movement promotion and broader lifestyle counselling alongside culturally adapted MedLife education. In contexts marked by higher psychological burden and less favourable sleep indicators, such as Algeria, integrated strategies combining lifestyle promotion with sleep-health and mental wellbeing support may be particularly relevant. In countries where social participation appeared comparatively lower, including some European contexts such as France, Germany, and Italy, public-health initiatives may benefit from incorporating community-based and socially engaging formats rather than focusing on dietary change alone. Conversely, countries displaying comparatively favourable profiles in selected domains, such as Spain for MedLife adherence or Tunisia for lower sedentary time and higher social participation, may provide useful contextual examples for strengths-based adaptation and knowledge transfer within the broader Mediterranean region.

Overall, these findings emphasize that effective Mediterranean lifestyle promotion should consider the broader behavioural and psychosocial ecosystem in which dietary practices are embedded. A nuanced understanding of country-specific lifestyle profiles may therefore contribute to more equitable and effective public-health strategies aimed at promoting holistic healthy lifestyles across diverse sociocultural contexts.

## 6 Future research directions

Future research should build on these findings by employing longitudinal designs to explore temporal relationships between lifestyle domains and psychosocial outcomes, as well as experimental and intervention studies to test the effectiveness of multidimensional Mediterranean lifestyle programs. In addition, person-centered analytical approaches such as cluster or latent profile analysis may help identify multidimensional lifestyle configurations at the individual level and complement the country-comparative perspective adopted in the present study. Further work is also needed to refine cross-cultural measurement tools, investigate structural, socioeconomic, and environmental determinants of lifestyle behaviours, and explore how digital technologies can be leveraged to support culturally adapted lifestyle interventions.

## Data Availability

The raw data supporting the conclusions of this article will be made available by the authors, without undue reservation.
