# Supplementary material for: Cross-country variability in Mediterranean lifestyle adherence and its psychosocial and behavioural correlates: insights from the MEDIET4ALL project across 10 Mediterranean and neighboring countries
Source: Front Nutr. 2026 Apr 15;13:1807414. doi: 10.3389/fnut.2026.1807414 (PMC13127259; doi:10.3389/fnut.2026.1807414)
Supplement: Supplementary file 1 [file Image_1.pdf]

Supplementary file

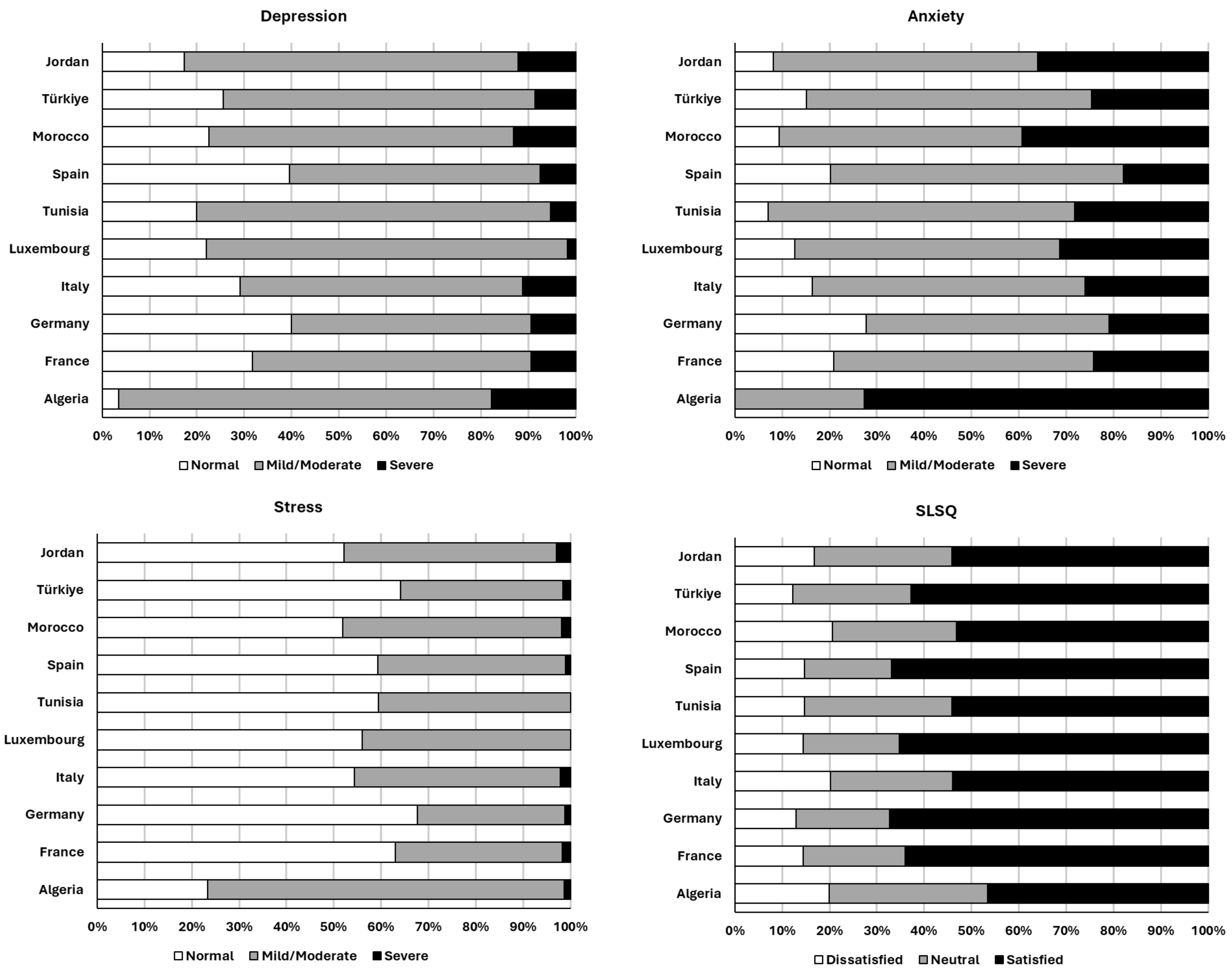

Figure S1: Distribution of categorical outcomes for psychological distress and life satisfaction's (SLSQ) across countries.

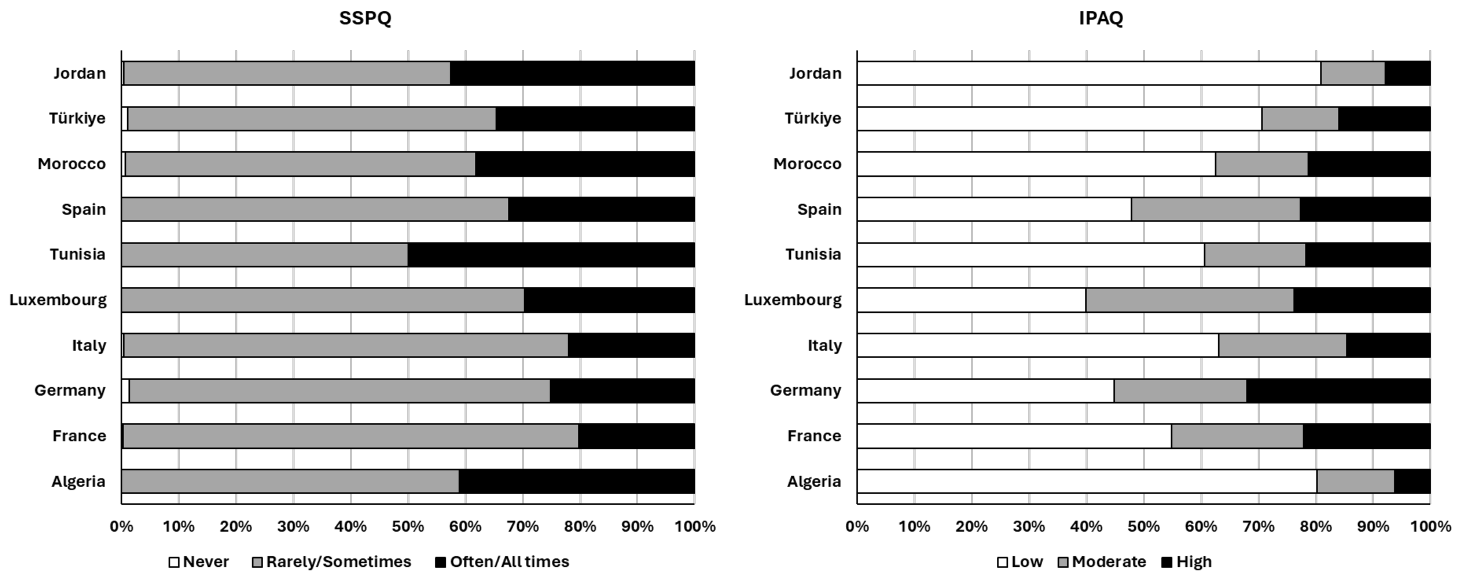

Figure S2: Distribution of categorical outcomes for social participation and physical activity across countries.

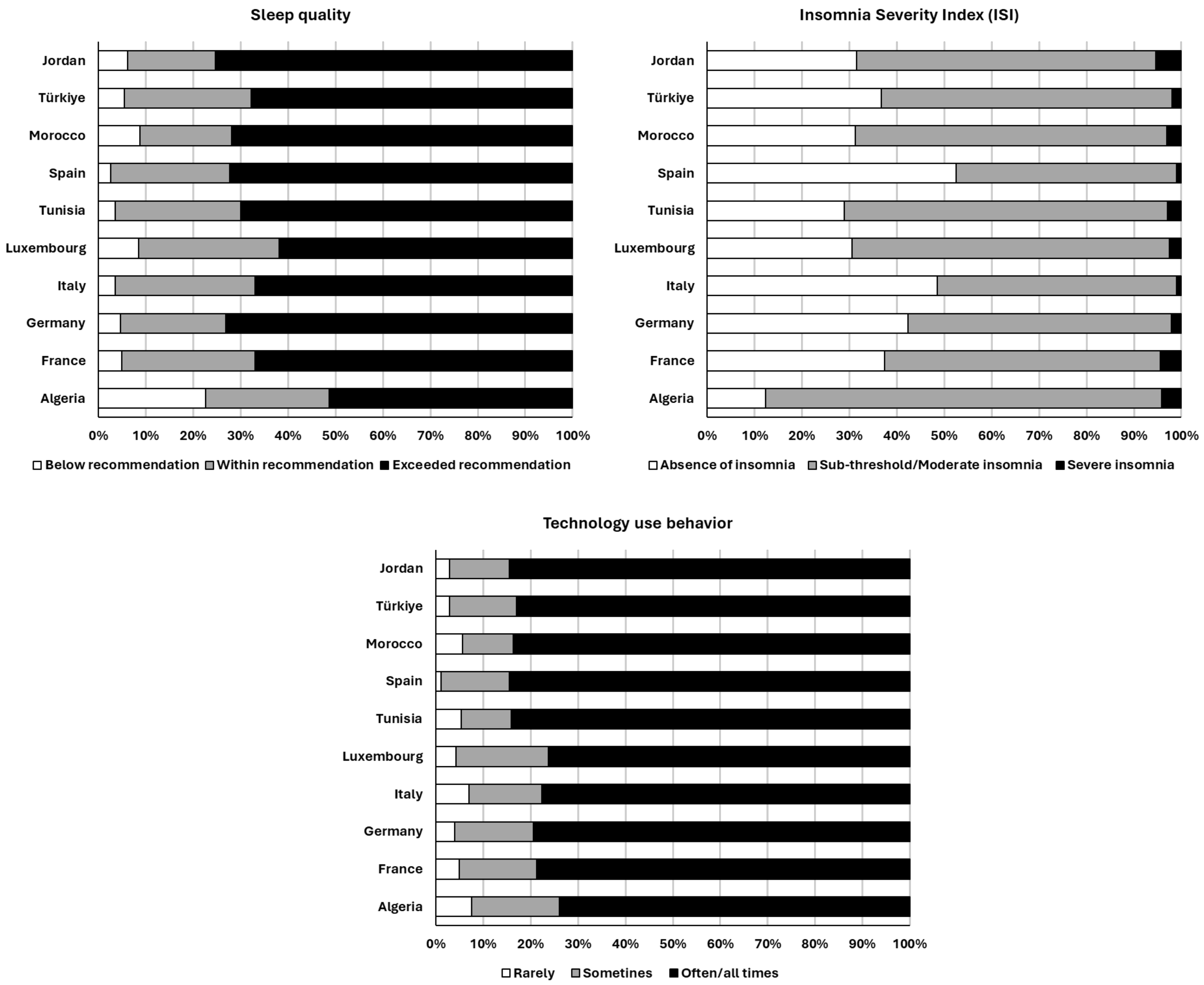

Figure S3: Distribution of categorical outcomes for sleep quality, insomnia, and technology use across countries.
